# A case of necrotizing fasciitis caused by group G streptococcus following a single dose of dose-dense doxorubicin and cyclophosphamide in a breast cancer patient

**DOI:** 10.1093/omcr/omag006

**Published:** 2026-02-24

**Authors:** Ayu Ajitomi, Akihiko Ozaki, Hideki Tayama, Norihiro Kido, Ryoko Murao, Yoshitaro Yoshida, Takanori Asakura, Toyoaki Sawano, Tomohiro Kurokawa, Kenji Gonda, Mika Nashimoto, Masahiro Wada, Kazunoshin Tachibana, Atsushi Koyama, Hiroaki Shimmura

**Affiliations:** Breast and Thyroid Center, Jyoban Hospital of Tokiwa Foundation, Iwaki City, Fukushima 972-8322, Japan; Breast and Thyroid Center, Jyoban Hospital of Tokiwa Foundation, Iwaki City, Fukushima 972-8322, Japan; Emergency and Critical Care Center, Iwaki City Medical Center, Iwaki City, Fukushima 973-8402, Japan; Emergency and Critical Care Center, Iwaki City Medical Center, Iwaki City, Fukushima 973-8402, Japan; Emergency and Critical Care Center, Iwaki City Medical Center, Iwaki City, Fukushima 973-8402, Japan; Emergency and Critical Care Center, Iwaki City Medical Center, Iwaki City, Fukushima 973-8402, Japan; Department of Respiratory Medicine, Kitasato University Kitasato Institute Hospital, Tokyo 108-8642, Japan; Department of Clinical Medicine (Laboratory of Bioregulatory Medicine), Kitasato University School of Pharmacy, Tokyo 108-8641, Japan; Department of Surgery, Jyoban Hospital of Tokiwa Foundation, Iwaki City, Fukushima 972-8322, Japan; Department of Surgery, Jyoban Hospital of Tokiwa Foundation, Iwaki City, Fukushima 972-8322, Japan; Breast and Thyroid Center, Jyoban Hospital of Tokiwa Foundation, Iwaki City, Fukushima 972-8322, Japan; Kameda Medical Center Breast Center, Kamogawa City, Chiba 296-0041, Japan; Breast and Thyroid Center, Jyoban Hospital of Tokiwa Foundation, Iwaki City, Fukushima 972-8322, Japan; Department of Breast Surgery, Utsunomiya Central Clinic, Utsunomiya City, Tochigi 321-0112, Japan; Breast and Thyroid Center, Jyoban Hospital of Tokiwa Foundation, Iwaki City, Fukushima 972-8322, Japan; Department of Breast Surgery, Fukushima Medical University, Fukushima City, Fukushima 960-1295, Japan; Emergency and Critical Care Center, Iwaki City Medical Center, Iwaki City, Fukushima 973-8402, Japan; Department of Urology, Jyoban Hospital of Tokiwa Foundation, Iwaki City, Fukushima 972-8322, Japan

**Keywords:** fasciitis, necrotizing, streptococcal infections, breast neoplasms, antineoplastic combined chemotherapy protocols, drug-related side effects and adverse reactions

## Abstract

Necrotizing fasciitis (NF) is a rapidly progressive and life-threatening soft tissue infection. Although it is known to occur in immunocompromised patients, NF following a single dose of chemotherapy has rarely been reported. We describe a 53-year-old woman with hormone receptor-positive, HER2-negative breast cancer and newly diagnosed type 2 diabetes mellitus who developed NF eight days after receiving her first cycle of dose-dense doxorubicin and cyclophosphamide. Initially mistaken for a drug eruption, her condition deteriorated rapidly despite surgical intervention and intensive care. She died of septic shock, and Group G Streptococcus was identified from blood cultures. This case highlights the need to consider NF in post-chemotherapy patients presenting with erythema and systemic symptoms, even after a single dose, particularly when risk factors such as diabetes and obesity are present. Prompt recognition and intervention remain essential to improve survival.

## Introduction

Necrotizing fasciitis (NF) are surgical emergencies characterized by rapidly progressive soft-tissue necrosis and associated with significant morbidity and mortality, with reported mortality rates ranging from 6% to 76% [1]. Active malignancy, particularly in patients undergoing chemotherapy, has been noted to confer higher risk, with several case reports documented in the literature. However, the timing of chemotherapy in relation to NF onset is often unreported [2, 3] or described after multiple cycles of chemotherapy [4, 5].

## Case report

A 53-year-old female patient with obesity (Body Mass Index 38 kg/m^2^) presented with a right breast mass 71 days before chemotherapy (X-71). On X-66, she was diagnosed with T2N0M0, hormone-positive, human epidermal growth factor receptor 2 (HER2)-negative invasive lobular carcinoma of the right breast with a Ki67 index of 15%. Concurrently, she was diagnosed with type 2 diabetes mellitus (Hemoglobin A1c 7.8%) and prescribed metformin 1000 mg daily.

She underwent right total mastectomy with axillary lymph node dissection 29 days before starting chemotherapy. On X + 0, the first cycle of dose-dense doxorubicin (60 mg/m^2^) and cyclophosphamide (600 mg/m^2^) (ddAC) therapy was initiated. Pre-chemotherapy laboratory results were unremarkable.

On X + 1, the patient reported only oral discomfort during her outpatient visit. On X + 6, the patient presented with nausea and a rash on her right lower extremity. The latter was presumptively diagnosed as a drug eruption and treated with topical corticosteroids.

**Figure 1:**
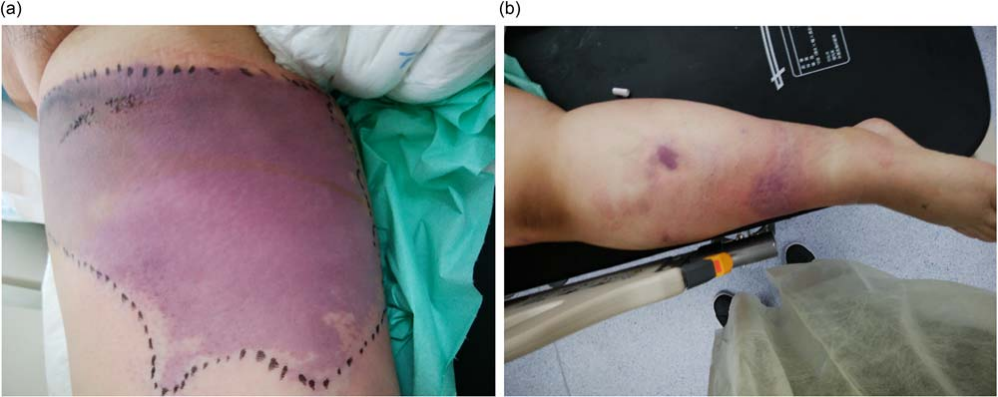
(a) Geographic erythematous lesion on the right thigh and (b) on the right lower leg observed 8 days after a single cycle of dose-dense doxorubicin–cyclophosphamide chemotherapy.

On X + 8, the patient returned with marked swelling, erythema, and coolness of the right lower extremity. On examination, her vital signs were: Heart Rate 150 bpm, Blood Pressure 100/80 mmHg, Body Temperature 38.5 degree Celsius, Oxygen Saturation 96% on room air. A geographic erythematous lesion was observed on the right thigh (Fig. 1a) and right leg (Fig. 1b).

Laboratory results showed: Creatine Kinase 862 U/l, Blood Urea Nitrogen 45.4 mg/dl, Creatinine 4.48 mg/dl, C-Reactive Protein 48.0 mg/l, Na 131 mEq/l, White Blood Cell count 500/μl, Hemoglobin 11.0 g/dl, Platelet count 40 000/μl. The patient was emergently admitted. Blood cultures obtained on admission later yielded Group G beta-hemolytic streptococcus. Suspecting NF of the right lower extremity, the patient was transferred to a tertiary care center.

Upon transfer, computed tomography demonstrated that the infection had already extended proximally beyond the hip joint into the retroperitoneal space. Because limited emergent debridement was unlikely to achieve adequate source control, and the patient exhibited severe neutropenia, thrombocytopenia, and organ dysfunction, immediate surgery under general anesthesia was considered extremely high-risk. Therefore, percutaneous drainage and infection assessment were prioritized during the first four days after transfer, followed by staged surgical debridement on X + 11 and X + 26.

On X + 11, a muscle biopsy was performed. Although histological examination did not reveal overt necrosis, the widespread infiltration of inflammatory cells made it impossible to definitively exclude necrotizing fasciitis. These findings suggested that, by day 8 (at the time of transfer), the infection had already extended proximally beyond the hip joint into the retroperitoneal space. Surgical debridement under general anesthesia was performed on X + 11 and again on X + 26.

The patient initially showed improvement in septic shock and was weaned off vasopressors. However, her condition gradually deteriorated, becoming refractory to vasopressors, and she ultimately succumbed on X + 70, 63 days after transfer.

## Discussion

This case describes an obese female patient with newly diagnosed type 2 diabetes mellitus who developed NF following a single dose of ddAC as adjuvant chemotherapy for breast cancer. While the association between chemotherapy and NF has been documented in the literature [1–5], reports of onset after a single chemotherapy dose are scarce. This case underscores the potential for rapid onset and fatal progression when multiple risk factors coexist.

The patient had previously been misdiagnosed with a drug eruption, delaying appropriate intervention. Drug eruptions typically appear bilateral with well-defined borders. In contrast, cellulitis presents as unilateral erythema with warmth, while necrotizing fasciitis is unilateral with non-blanching purpura, blisters, and variable temperature. This non-blanching characteristic is key for identifying NF.

In this case, the Laboratory Risk Indicator for Necrotizing Fasciitis score was 10 on the transfer day [6]. While initial skin findings alone made NF difficult to suspect, earlier laboratory testing could have facilitated diagnosis, enabling timely surgery.

In addition, immediate surgical debridement was not performed upon transfer due to several clinical considerations, including radiologic evidence of retroperitoneal extension, identification of Group G rather than Group A Streptococcus, and the presence of severe cytopenia and organ dysfunction, all of which made emergent surgery high-risk.

Importantly, no clear primary focus of infection was identified, and the clinical course did not allow a clear distinction between a localized soft-tissue infection and a systemic process driven by chemotherapy-induced immunosuppression. It is therefore plausible that the infection may have initially been localized but progressed uncontrollably due to the combined effects of immunosuppression, diabetes, and obesity, rather than behaving as a typical soft-tissue infection in an otherwise immunocompetent patient.

While early and complete debridement remains the standard recommendation for NF, this case highlights the clinical challenge of balancing surgical urgency with physiological stabilization in patients with extensive disease and severe immunosuppression. In this context, the present case underscores the need for heightened clinical suspicion and early multidisciplinary assessment in post-chemotherapy patients presenting with atypical or rapidly progressive soft-tissue infections, even when an obvious primary focus is absent.
